# From mechanistic models to artificial intelligence: exploring the potential of digital twins in geriatric oncology

**DOI:** 10.3389/frai.2026.1760912

**Published:** 2026-07-23

**Authors:** Panagiotis Karampelesis, Spyros Denazis, Odysseas Koufopavlou, Evangelia I. Zacharaki

**Affiliations:** 1 Department of Electrical and Computer Engineering, University of Patras, Patras, Greece; 2 Department of Computer Engineering and Informatics, University of Patras, Patras, Greece

**Keywords:** artificial intelligence, breast cancer, digital twins, frailty, geriatric oncology, machine learning, mechanistic models

## Abstract

This survey explores how machine learning and artificial intelligence (AI) can be integrated with mechanistic models to create more accurate, dynamic, predictive, and personalized representations of biological systems, commonly referred to as digital twins (DTs). Mechanistic models, such as pathway-based Boolean or differential equation frameworks, provide interpretable insights into biological processes; however, calibrating these models to represent individual variability across large, heterogeneous cohorts remains a significant challenge, as their physically constrained structures often lack the flexibility to capture complex, non-mechanistic nuances in patient data. Focusing on elderly cancer patients–a vulnerable population underrepresented in clinical research–we discuss how hybrid DTs can bridge the gap between interpretable mechanistic frameworks and flexible, predictive AI approaches, enabling continuous monitoring, risk stratification, and adaptive treatment planning. To illustrate these principles, we present a proof-of-concept case study involving a synthetic breast cancer dataset in which comprehensive geriatric assessment, clinical tests, and quality of life measures inform dosing decisions for older patients via a Markov Decision Process. By combining a synthesis of current literature with the application of a sequential decision-making framework optimized using longitudinal data, this work provides a foundational understanding for researchers and clinicians interested in leveraging DTs to improve personalization and outcomes in geriatric oncology.

## 1 Introduction

Digital twin (DT) technology in healthcare has gained significant attention over the last decade. A DT is a virtual representation of the patient that enables physicians and scientists to explore potential outcomes of different treatments (Katsoulakis et al., 2024). When combined with individual patient characteristics, DTs can revolutionize personalized therapy by enabling tailored approaches to treatment. Moreover, by integrating real-time data from wearables and medical devices, DTs can continuously monitor patient progress and dynamically adapt treatment strategies.

DTs rely on two primary approaches to model and simulate complex systems: mechanistic models, which use fundamental scientific principles and mathematical equations to represent system behavior, and artificial intelligence (AI) and machine learning (ML) methods, which analyze big datasets to identify patterns and make predictions. Mechanistic models are based on established principles, can be tailored to specific biological or physical processes, and offer transparency and interpretability. However, they often struggle to capture cross-scale interactions and to account for the variability in holistic models that can guide therapy (Procopio et al., 2023).

By contrast, AI/ML methods are emerging as transformative DT technologies that can handle complex, non-linear systems without predefined rules. AI enhances predictive capability by integrating heterogeneous data sources to identify correlations and patterns that may not be evident through traditional modeling, while real-time data integration allows for continuous adaptation and refinement of DT models. In geriatric oncology, AI/ML models have been used to predict treatment response, stratify patients based on frailty or comorbidities, and estimate risks of adverse events (Shirini et al., 2023). Importantly, they offer opportunities to combine biological, clinical, and imaging-derived features with geriatric assessments into holistic models that can guide therapy.

While there is a vast amount of works investigating the use of DTs for the delivery of optimized cancer treatment, the unique needs of older patients are rarely taken into consideration (Pilleron and O'Hanlon, 2023). The biological effects of aging, including cell senescence, the accumulation of random genetic mutations over time, and epigenetic changes, lead to reduced organ functionality and a greater likelihood of developing chronic conditions. In addition, low-level chronic inflammation increases susceptibility to age-related diseases. This increased vulnerability to adverse health outcomes is summarized under the term *frailty*. Frailty indexes are now considered essential for risk stratification, as they provide more accurate prognostic information than chronological age alone (Xue, 2011; Guerard et al., 2017). However, despite their clinical importance, frailty measures remain underutilized in computational modeling frameworks of biological systems. DTs of older patients can provide a robust framework for modeling individual health trajectories and predicting treatment responses in aging populations.

The objective of this survey is to explore how AI/ML applications can be integrated with mechanistic modeling approaches on different scales to develop robust DTs of complex biological processes involved in cancer and aging. Comprehensive geriatric assessment (CGA), which evaluates domains such as physical function, comorbidities, cognition, mood, nutrition, and social support (Welsh et al., 2014), plays a crucial role in identifying frailty and predicting health outcomes in older patients. ML models using CGA data offer a pathway to enhance treatment decisions by assessing frailty and predicting which patients are likely to respond better to less aggressive treatment. DTs can be used to simulate risks of treatment-related toxicity, such as chemotherapy toxicity from targeted therapies in frail patients, and to adapt therapy plans to balance efficacy with quality of life (QoL). For example, ML predictive models might recommend a shorter therapy duration for frail patients to minimize side effects while maintaining treatment efficacy.

The paper first outlines the hallmarks of cancer and aging that have been incorporated into computational frameworks. We then outline existing platforms and tools that apply mechanistic models in oncology and aging research, followed by a discussion of AI/ML applications that support the development of DTs and guide clinical decision-making in elderly cancer patients. To demonstrate the practical utility of the discussed frameworks, we include a synthetic breast cancer dataset and a Markov Decision Process (MDP) simulation as an illustrative example of personalized treatment strategies (Singh et al., 2024; Imani et al., 2020; Bennett and Hauser, 2013; Schaefer et al., 2005). Our aim is to explore the utility of DT technologies in modeling frailty progression and informing treatment decisions for older cancer patients. Enabling technologies, including sensors (self-tracking tools, smart devices), Internet-Of-Things, cloud computing, virtual or extended reality, for the purpose of digital healthcare assistance (Abdollahi et al., 2024), are out of the scope of the current survey.

## 2 Literature review

### 2.1 Mechanistic modeling

Mechanistic models are explicit mathematical representations of biological systems that dissert molecular, cellular, or system-level interactions. These approaches prioritize the construction of models based on detailed, well-characterized mechanisms. Unlike data-driven models that infer patterns from large datasets, mechanistic approaches emphasize hypothesis-driven analysis, enabling researchers to simulate and predict system responses under various conditions. Mechanistic models often simulate how biological systems respond to external stimuli or perturbations, such as drug treatments and changes in nutrient availability, or predict the effects of biological processes in acute or chronic conditions. Common mechanistic modeling families include equation-based models, constraint-based models, network- or rule-based models, agent-based models (ABMs), system dynamics models, and stochastic mechanistic simulations. These approaches differ in mathematical formulation and biological scale, but they share the objective of representing explicit biological, physiological, or physical mechanisms.

Several mechanistic frameworks have been widely applied in biomedical research (Procopio et al., 2023), offering valuable tools for modeling complex physiological changes. In the context of aging and cancer, such models have been used to simulate immune decline, tumor progression, and treatment response (Berben et al., 2021; George and Levine, 2018; Lorenzo et al., 2024; Qian et al., 2024). Equation-based models, such as ordinary differential equations (ODEs) and partial differential equations (PDEs), have been used to describe dynamic changes in molecular concentrations—such as cytokine levels or drug pharmacokinetics, which are often dysregulated in older cancer patients due to immunosenescence and altered metabolism (Lefèvre et al., 2025; Shive and Pandiyan, 2022). Additionally, discrete-time delay ordinary difference equations have been utilized to model the stability of maintenance chemotherapy and detect minimal residual disease (Majumder, 2023). Constraint-based models (e.g., Flux Balance Analysis) estimate feasible metabolic flux distributions under biomedical and stoichiometric constraints, making them particularly useful for capturing age-related shifts in cellular energetics and tumor metabolism (Meeson and Schwartz, 2024). Network models, including Boolean and rule-based approaches, can represent signaling or gene-regulatory interactions that are frequently disrupted in both aging tissues and malignancies, such as p53 pathway alterations or chronic inflammation (Ge and Qian, 2009; Gudkov and Komarova, 2016). Stochastic mechanistic models, such as the Gillespie stochastic simulation algorithm (SSA), extend these representations by incorporating randomness into reaction dynamics, which is particularly important in systems with few molecules (Clermont et al., 2007). The incorporation of molecular noise and variability–features becomes increasingly relevant in aged systems where cellular heterogeneity and damage accumulation are pronounced (Martinez-Jimenez et al., 2017).

ABMs simulate the actions and interactions of individual agents (e.g., patients, cells) to assess their effects on the system as a whole. ABMs are useful for capturing complex, dynamic systems. They differ fundamentally from STMs, Markov Models, and DES, which are more aggregate or process-focused.

System dynamics models (Cassidy et al., 2019; Chodera and Noé, 2014) use flows, continuous feedback loops, and time delays (rather than discrete states or events) to simulate the behavior of complex systems over time. They are often used in public health and policy analysis.

Global (i.e., population-level) models can be tailored by incorporating patient-specific data such as gene expression profiles, medical imaging (e.g., MRI, mammography), and frailty scores from geriatric tests. This integration enhances the precision and applicability of models, supporting personalized digital twin applications capable of accurately predicting individualized patient responses.

#### 2.1.1 Databases and tools for modeling biological processes

Turning biological knowledge into computational models is rarely a straightforward process. This process begins with gathering relevant biological data, such as gene expression profiles, protein-protein interactions, and signaling pathways, and ends with the construction of executable models capable of simulating complex biological behaviors under different conditions, such as disease progression or response to treatments. Although the exact steps may vary depending on the specific study, there is a set of commonly used resources, tools, and practices that establish an informal roadmap guiding the transition from biological data to computational model generation.

Initially, the process involves collecting biological data from specialized and curated databases. Resources such as KEGG (Kanehisa et al., 2024) and Reactome (Milacic et al., 2023) offer comprehensive pathway data, while interaction databases like STRING (Szklarczyk et al., 2022) and BioGRID (Oughtred et al., 2021) provide molecular interaction networks. These collected datasets may require editing and visualization, a crucial step to facilitate comprehension and subsequent analysis. Visualization tools [e.g. CellDesigner (Funahashi et al., 2003)] enable the construction of structured pathway maps in standardized formats, such as the Systems Biology Markup Language (SBML) (Hucka et al., 2015), while broader network analyses, including the integration of diverse omics datasets, can be supported by tools like Cytoscape (Shannon et al., 2003).

Following the visualization, the static maps must be converted into dynamic computational models that can simulate biological behavior. Platforms, such as CasQ (Aghamiri et al., 2020), bridge this gap by translating CellDesigner (Funahashi et al., 2003) pathway maps into executable Boolean or rule-based formats, facilitating further exploration with specialized simulation platforms, including GINsim (Gonzalez et al., 2006), BioLQM (Naldi, 2018), and CoLoMoTo (Naldi et al., 2015). For capturing time-dependent behaviors—which is critical when modeling scenarios like disease progression or treatment response over extended period—ODE-based modeling is commonly applied using specialized software like COPASI (Hoops et al., 2006) or computational environments like MATLAB. In scenarios where stochastic variability is significant, such as modeling molecular fluctuations and probabilistic signaling events, with platforms like MaBoSS (Stoll et al., 2012) catering specifically to these requirements. Additionally, web-based environments, such as Cell Collective (Helikar et al., 2012), serve as accessible and collaborative platforms, enabling rapid testing, iterative refinement, and sharing of biological models.

The above resources are instrumental in modeling the intracellular and molecular layers of a DT as they can simulate internal biological states that are not directly measurable. Mechanistic biomarkers extracted with such tools provide the biological context necessary for subsequent stochastic decision-making frameworks to optimize decisions beyond simple clinical observation.

### 2.2 AI/ML techniques

In this section, we are reviewing research trends and key AI/ML concepts in biological systems for DT applications. Machine learning is a branch of artificial intelligence that allows computers to learn from data and make decisions without requiring explicit programming (Jordan and Mitchell, 2015). AI is being increasingly used in biology, medicine, and healthcare to analyze biological systems, predict disease progression, or optimize patient care (Deng et al., 2022). It is applied in different scales, from molecular-level data to population-wide health information. Machine learning techniques can be divided into three types depending on how the learning happens: supervised learning, unsupervised learning, and reinforcement learning. In the next subsections, we briefly describe the differences of each ML type and discuss examples of potential relevance in geriatric oncology.

#### 2.2.1 Supervised learning for prediction modeling

Supervised learning methods underpin the development of clinical prediction models that estimate patient-specific outcomes based on past data. These models learn from labeled datasets–cases where both patient characteristics and clinical outcomes are known–to forecast what might happen in new cases. Typical supervised tasks include classification, which involves assigning inputs to discrete categories (e.g., determining whether a tumor is malignant or benign), and regression, which focuses on predicting continuous numerical values (e.g., estimating time to disease progression).

In cancer care for older adults, supervised learning models are commonly used to estimate the risk of clinically relevant outcomes based on structured patient data. Such outcomes may include treatment-related toxicity, early mortality, hospital readmission, or functional decline—factors that are especially critical when managing frail or vulnerable patients (Hurria et al., 2011). Structured data sources often include electronic health records, laboratory values, imaging-derived features, and clinical assessment scores.

By learning from historical cases where the true outcome is known, these models can predict how a new patient might respond to a given therapy. This predictive information is then used to support clinical decisions, such as whether to adjust the treatment dose, switch regimens, or initiate supportive care earlier (Hurria et al., 2011; Chan et al., 2025). Commonly used algorithms include logistic regression, random forests, and gradient boosting machines, which are favored for their interpretability and predictive accuracy in clinical settings.

In digital twin applications, supervised models serve as components that simulate outcome trajectories under different interventions, allowing for proactive adaptation of therapy over time. This is particularly useful in older patients, where standard treatment protocols may not adequately account for physiological heterogeneity, comorbidities, or dynamic changes in frailty status (Sena et al., 2019; Chu et al., 2023; Fabris et al., 2017). By integrating such models into digital twin frameworks, clinicians can forecast individualized outcomes across multiple treatment pathways, proactively adapting care in ways that are otherwise infeasible in traditional one-size-fits-all protocols (Heudel et al., 2025).

#### 2.2.2 Unsupervised learning for pattern discovery

In contrast to supervised learning, which requires known outcomes, unsupervised learning focuses on discovering hidden patterns within unlabeled data. This makes it particularly powerful during the early phases of data exploration, when the goal is not to predict but to understand the structure and relationships within complex datasets. Two of the most common applications include clustering (grouping similar data instances) and dimensionality reduction (simplifying data while preserving core information).

In the context of geriatric oncology, where patients exhibit wide variability in comorbidities, functional status, and treatment tolerance, unsupervised learning methods can reveal natural groupings or subtypes within the patient population. For instance, clustering techniques such as K-means or hierarchical clustering have been used to cluster older adults with cancer into latent risk profiles based on patterns in symptoms, lab values, or frailty scores—even without predefined clinical outcomes (Lopez et al., 2018). Studies have shown that patients belonging to certain symptom clusters (e.g., high fatigue, poor appetite, pain) are at significantly greater risk of adverse events, including emergency visits, early functional decline, or premature mortality (Xu et al., 2023). Unsupervised learning can uncover clinically meaningful patterns in data even when those patterns have not yet been linked to specific endpoints. For example, these techniques can help clinicians identify under-recognized subgroups—such as patients with silent functional vulnerabilities or those at high psychosocial risk—that may benefit from early intervention or individualized care plans (Golzy et al., 2023).

Moreover, dimensionality reduction techniques like Principal Component Analysis (PCA) or t-distributed Stochastic Neighbor Embedding (t-SNE) can help visualize patient distributions across high-dimensional datasets (Arjmand et al., 2022). This makes them especially useful for multi-omics integration (Arjmand et al., 2022; Vahabi and Michailidis, 2022), imaging data exploration, or summarizing large-scale geriatric assessment metrics. In digital twin development, these tools can assist in preprocessing steps by identifying core axes of variation and simplifying the model space without sacrificing key information.

By providing a deeper understanding of the internal structure of data, unsupervised learning supports data-driven personalization before any prediction is made. It allows clinicians and researchers to refine their hypotheses, define more targeted intervention strategies, and build more realistic digital twin cohorts grounded in the true complexity of aging and cancer heterogeneity.

#### 2.2.3 Stochastic decision-support and reinforcement learning for adaptive decision-making

Reinforcement learning (RL) is a machine learning paradigm in which an agent learns optimal actions through continuous interaction with an environment. Rather than relying on predefined outcomes or labeled datasets, the agent starts without prior knowledge and learns from experience, using trial and error to discover which actions maximize cumulative rewards (Mashayekhi et al., 2024). Feedback comes in the form of penalties or rewards, reinforcing actions that lead to better results and discouraging suboptimal ones. This makes RL particularly well-suited for dynamic, sequential decision-making tasks where outcomes unfold over time and adaptivity is critical.

In the context of cancer care, reinforcement learning can be applied to adaptive treatment planning, where decisions must evolve in response to how a patient reacts during therapy. For example, an RL-based model can be trained to manage the administration of CDK4/6 inhibitors or endocrine therapy, continuously adjusting dosage or scheduling based on evolving patient signals—such as changes in lab values, clinical scores, symptom severity, or early side effects. Each action taken by the model (e.g., continue, reduce, pause, or switch therapy) is evaluated based on its clinical consequences (Eastman et al., 2021), such as maintaining function, avoiding hospitalizations, or improving QoL. The model learns to prioritize actions that lead to better long-term outcomes, making it a valuable assistant for real-time clinical adaptation (Tardini et al., 2022). Moreover, RLs' ability to learn autonomously, without explicit supervision, is especially relevant in geriatric oncology, where responses to treatment are highly individualized, and clinicians must often make sequential decisions under uncertainty. By simulating thousands of possible patient trajectories, RL agents can uncover optimal treatment policies tailored to frailty levels, comorbidities, or changing risk profiles.

Within digital twin frameworks, reinforcement learning enables the simulation of multiple future treatment paths under varying patient states (Wentzel et al., 2024). It allows virtual agents to “test” decisions across a digital representation of the patient and determine which trajectory offers the best balance of safety and efficacy (Ghislain et al., 2025). For example, an RL-powered digital twin could explore how delaying chemotherapy by two weeks in a frail patient with low neutrophil counts might affect outcomes, learning from simulated consequences to refine future recommendations. As a result, reinforcement learning may support the automation of personalized, adaptive decision-making–helping clinicians move beyond static guidelines and embrace a dynamic, data-driven approach to geriatric cancer care (Bozcuk et al., 2025).

While reinforcement learning provides the algorithmic framework for learning adaptive clinical policies, its execution relies on stochastic decision-support methods–such as Markov Decision Processes (MDPs), state-transition models (STMs), and discrete event simulations (DES)—to mathematically define the uncertain patient states and virtual environments required for decision-making. STMs conceptualize clinical decision problems in terms of a set of states and transitions among these states (Siebert et al., 2012; Chandler et al., 2018). They are widely used in clinical decision analysis, health technology assessment, and economic evaluation, and can be implemented either as cohort-based or individual-based (first-order Monte Carlo) microsimulations. Markov models are a special case of STMs, assuming the Markov property, whereby transitions depend only on the current state rather than the full history. They are particularly useful for modeling chronic diseases and long-term outcomes. DES emphasizes the timing and sequencing of events, making it more process-oriented and commonly used in healthcare to simulate patient flow and resource utilization. Markov Decision Process extend this state-transition logic by incorporating actions and rewards, thereby providing a standard mathematical formulation for sequential decision-making and many model-based reinforcement learning approaches. When transition probabilities and reward functions are estimated or calibrated from data, MDPs can be interpreted as data-informed, model-based decision-support frameworks.

**Figure 1:**
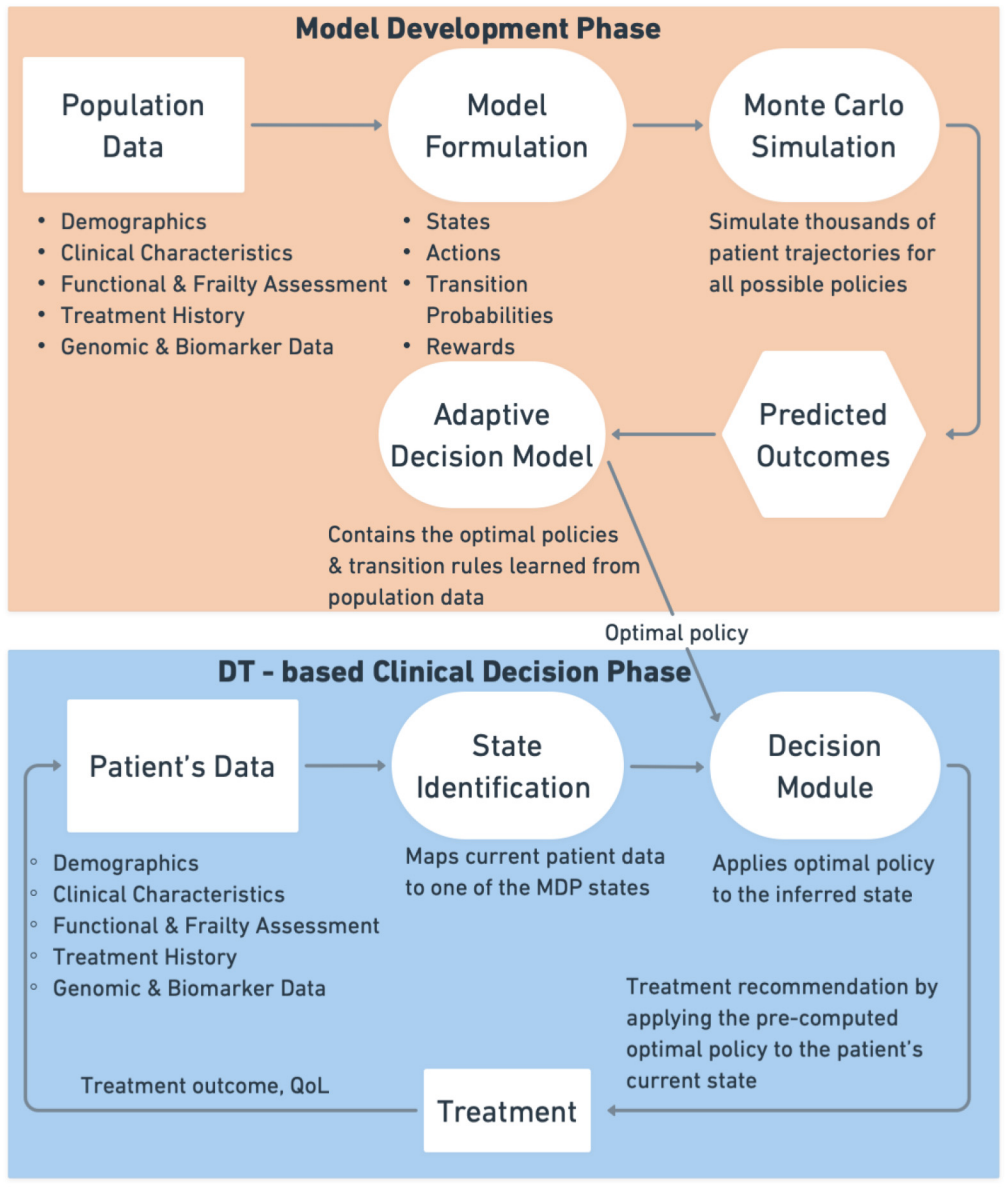
Workflow for development and application of a dynamic DT-based clinical decision support system. Population-level data that include per-visit patient profiles (*states*) and clinical choices (*actions*), such as escalate/de-escalate or maintain dose, are integrated into an adaptive model along with treatment outcomes. In the model development phase, data-driven probabilities are estimated for transitions among patient states defined by frailty status, clinical profile, and dose level, given actions and patient features. In the prediction phase, patient data are continuously integrated into the calibrated model to maintain an up-to-date DT of the patient. The DT enables real-time simulation of patient responses to alternative treatment strategies, with the inferred policy rules supporting treatment optimization.

In the present work, a MDP was incorporated as a probabilistic decision-support layer for sequential clinical decision-making. Treatment decisions are represented as actions selected according to the current patient state, transition probabilities, and rewards. Figure 1 illustrates how such probabilistic frameworks can be used for personalized treatment of older cancer patients. The workflow outlines both (i) the model development phase, where population-level data are used to estimate transition probabilities, rewards, and policy rules, and (ii) the prediction phase, where individual patient data are integrated into the calibrated model to maintain an up-to-date digital representation of the patient for sequential treatment optimization.

### 2.3 Synergy of mechanistic and AI/ML models

Mechanistic and AI/ML approaches have complementary strengths that can be leveraged to create digital twins capable of both interpretability and predictive accuracy. Mechanistic models capture causal biological knowledge through explicit equations or networks, which makes them transparent and clinically interpretable. However, they often struggle with patient heterogeneity, missing parameters, and incomplete biological knowledge. On the other hand, AI/ML models excel at learning complex, nonlinear patterns from large-scale patient data, but often operate as “black boxes” with limited mechanistic grounding.

**Figure 2:**
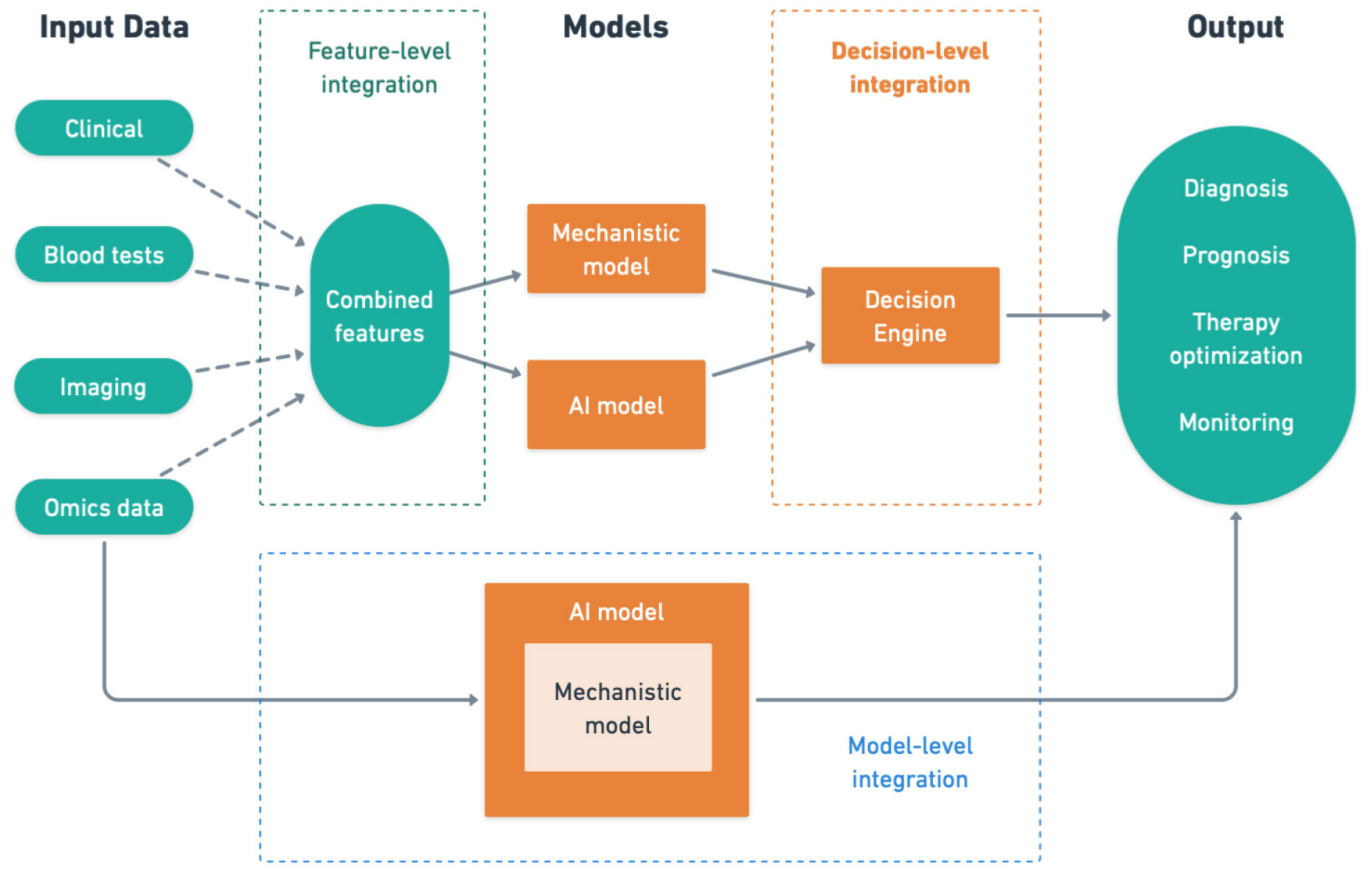
Integration of mechanistic and AI/ML models at different levels. At the feature level, heterogeneous patient data are combined before modeling. At the decision level, outputs from mechanistic and AI/ML models are synthesized by a decision engine to support clinical recommendations such as diagnosis, prognosis, therapy optimization, and monitoring. At the model level, mechanistic models are embedded within AI architectures, guided by biological priors to improve interpretability and biological plausibility.

Overall, knowledge integration may be applied at different levels (feature-level, decision-level, model-level) individually or in combination depending on the methodological framework, as illustrated in Figure 2. At the feature level, heterogeneous patient information is combined before modeling, ensuring that both mechanistic and data-driven methods are trained on richer and more informative inputs (Boehm et al., 2022; Tong et al., 2020). At the decision level, outputs from mechanistic and AI/ML models are synthesized by a decision engine, which balances the strengths of both approaches to provide clinically actionable recommendations (Othman et al., 2023). At the model level, mechanistic equations, such as ordinary or partial differential equations (ODEs/PDEs), are embedded directly within AI architectures, guiding the learning process with biological constraints and enabling interpretable predictions (Lagergren et al., 2020). Together, these three integration strategies form a flexible roadmap for building hybrid frameworks that combine interpretability and predictive accuracy, ultimately paving the way for robust and clinically relevant digital twin applications.

The model-level integration has led to several hybrid approaches that aim to combine the strengths of AI and mechanistic modeling: AI/ML can calibrate mechanistic models or identify features that improve their predictive power, while mechanistic priors help guide machine learning toward biologically meaningful solutions. For example, Mascheroni et al. (2021) developed a Bayesian framework that integrates tumor growth models with patient-specific data using ML, achieving more accurate forecasts of disease progression than either method alone.

Another influential approach is the concept of Physics-Informed Neural Networks (PINNs) introduced by Raissi et al. (2019). In this framework, deep neural networks are trained not only on available data but also under the constraints imposed by the governing partial differential equations of the system. By embedding physical laws into the learning process, PINNs merge mechanistic knowledge with data-driven flexibility. This allows for accurate solutions even with limited or noisy data, such as sparse observations and measurements affected by experimental uncertainty. This strategy has been applied successfully across domains such as fluid dynamics, quantum systems, and reaction-diffusion models, and illustrates how mechanistic structure can guide AI/ML toward biologically and physically meaningful predictions. Building on this idea, Lagergren et al. (2020) introduced Biologically-Informed Neural Networks (BINNs), which extend the PINN framework by incorporating biological constraints and learning unknown terms of mechanistic equations directly from experimental data. Applied to sparse and noisy in vitro cell migration assays, BINNs not only improved predictive accuracy but also uncovered a previously unrecognized delay in cell migration dynamics, demonstrating how hybrid approaches can both refine mechanistic models and reveal new biological insights (Lagergren et al., 2020).

Wu et al. (2022) demonstrated how integrating mechanism-based mathematical models with biomedical imaging can create clinically meaningful digital twins for oncology. Imaging provides patient-specific, non-invasive data on tumor morphology, vascularization, and cellularity, while mechanistic models translate these measurements into predictions of tumor growth and treatment response. For instance, diffusion-weighted and dynamic contrast-enhanced MRI have been used to calibrate reaction-diffusion models of breast cancer, enabling accurate forecasts of therapeutic outcomes under different regimens. This synergy illustrates how mechanistic transparency and AI-driven adaptability can be combined into holistic digital twins that support personalized decision-making in clinical oncology (Metzcar et al., 2024).

One clinically relevant application integrates AI/ML methods with physiologically based mechanistic models of cancer treatment, including quantitative systems pharmacology (QSP), physiologically based pharmacokinetic/pharmacodynamic (PBPK/PD), and QSP-IO frameworks (Sové et al., 2020; Wang et al., 2024). In these architectures, the mechanistic model provides a biologically interpretable backbone that represents processes such as drug exposure, tumor growth, immune-cell dynamics, signaling pathways, drug–target interactions, and treatment response. For example, QSP-IO frameworks can represent cancer-cell dynamics, antigen presentation, T-cell activation, immune checkpoints, and pharmacokinetics, enabling simulations of treatment response and virtual clinical trials. AI/ML methods can complement these frameworks by supporting parameter estimation, model calibration from multimodal and multi-omics data, surrogate modeling to reduce computational cost, uncertainty quantification, biomarker discovery, and patient stratification. In this way, hybrid mechanistic–AI models can move DTs beyond purely empirical associations or transition probabilities, allowing treatment-response predictions to be constrained by biological mechanisms and patient-specific data.

While hybrid mechanistic-AI models improve prediction and interpretability for cancer dynamics, a truly holistic digital twin must integrate additional layers of patient information and evolve continuously over time. Such a system should combine mechanistic disease models with AI/ML pipelines that process multimodal data—including imaging, genomics, electronic health records, wearable biosensors, and patient-reported outcomes–into an adaptive, patient-specific representation (Parvin et al., 2025). Recent work illustrates this direction. Dynamic digital twins are designed to continuously update with new clinical and molecular data, in a manner similar to how smart city twins adapt in real time (Karaman and Sebin, 2025). These frameworks move beyond static, one-time models toward adaptive systems that assimilate information over time. By integrating multimodal data streams, DTs can simulate alternative treatment scenarios, anticipate resistance or toxicity, and support proactive decision-making in clinical practice (Lyu et al., 2025; Rabinovici-Cohen et al., 2022).

## 3 Materials and methods

**Figure 3:**
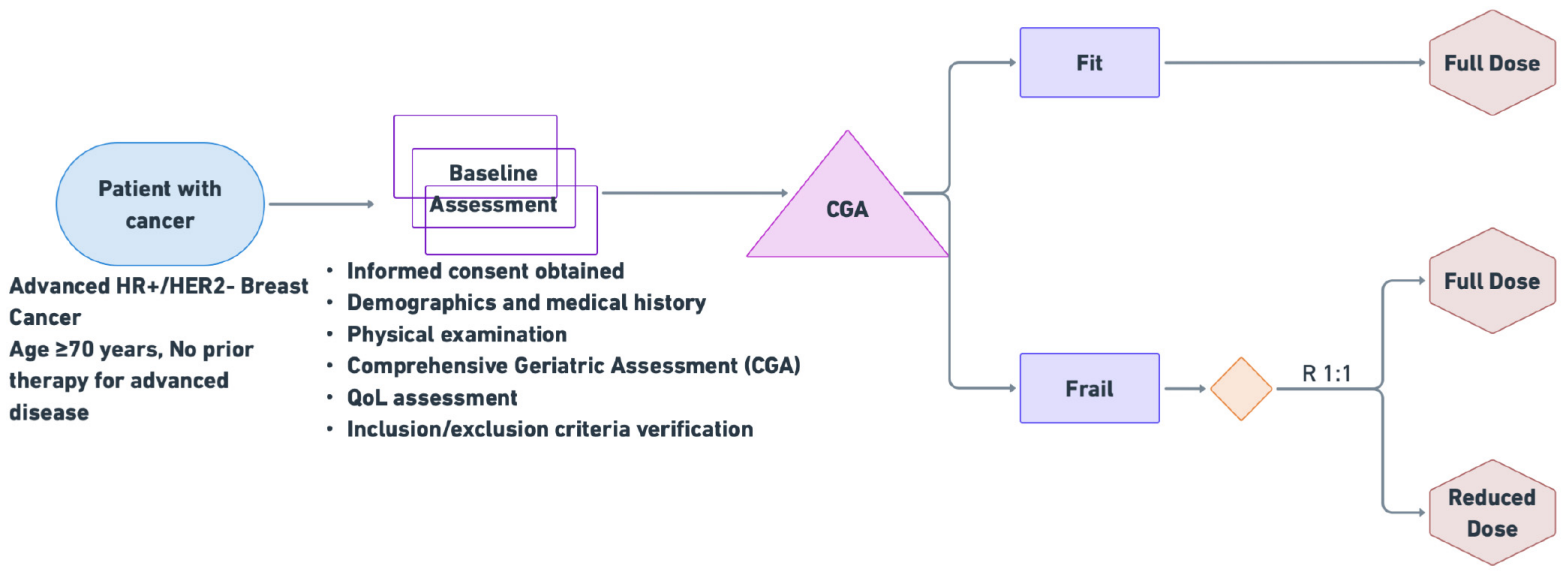
Following baseline assessment and CGA, patients are classified as fit or frail. Fit patients receive the full recommended dose of CDK4/6 inhibitor plus endocrine therapy. Frail patients are randomized (1:1) to either a full or a reduced dose. These branches represent the initial patient states that form the basis of the MDP framework.

To demonstrate how a probabilistic model could be used for DT-based clinical decision support in geriatric oncology, we use the objectives of a clinical study (IMPORTANT Project, 2023) as an application example, and a Markov Decision Process as a data modeling technique (Figure 1). The *IMPORTANT* study aims to investigate the prognosis, tolerability, and QoL of vulnerable or frail older breast cancer patients treated with lower a initial dose of CDK 4/6-inhibitors plus endocrine therapy compared to the recommended full dose of CDK 4/6-inhibitors. The rationale is that such a tailored treatment strategy reduces the risk for adverse events in such a vulnerable population. Comprehensive geriatric assessment is applied as the basis for the assessment of patients' frailty before randomization. To examine whether the frailty-based treatment adjustment is beneficial, patients are initially stratified into two groups: *fit*—receiving the recommended full dose of a CDK4/6 inhibitor in combination with endocrine therapy (ET)—or *vulnerable/frail*, in which case they are randomized in a 1:1 ratio to receive either the full dose or a reduced dose (one level below standard) of CDK4/6i plus ET, as shown in Figure 3.

Patients are followed longitudinally, and outcomes such as toxicity-related death, cancer recurrence, complete response, and death from cancer or other causes are recorded. Clinical evaluations occur at predefined intervals aligned with the clinical protocol, with dose-modification decisions occurring at each treatment cycle (approximately every 4 weeks), while efficacy and CGA assessments are updated every 3 and 6 months, respectively. Treatment continues until progression, unacceptable toxicity, or a decision to stop. Details on the study population and clinical trial design can be found in IMPORTANT Project (2023).

### 3.1 Case study

To demonstrate AI-driven dynamic dose optimization in a proof-of-concept case study, we pursued two methodological developments. First, we constructed a large-scale synthetic dataset designed to approximate the characteristics of the studied population and typical clinical outcomes. Second, we developed a computational framework based on a Markov Decision Process to model patient responses under alternative treatment strategies. While the MDP model presented here utilizes a synthetic dataset to illustrate the potential for adaptive dosing in breast cancer, we acknowledge that clinical implementation requires substantiation with high-fidelity, real-world data. Markovian transitions are sensitive to the historical distributions of patient outcomes. The framework proposed serves as a computational architecture currently being prepared for validation using data from the *IMPORTANT* project. This upcoming analysis will replace synthetic priors with real clinical observations, allowing for a transition from a proof-of-concept to a clinically validated digital twin.

The workflow for the development and application phases of the MDP is depicted in Figure 1. In clinical settings, quality-adjusted life years (QALY) are typically derived from patient-reported outcomes and QoL assessments. In the present study, QALY is used as a proxy reward function derived from simulated patient trajectories, designed to reflect the trade-off between treatment benefit and toxicity. The MDP is optimized by computing an optimal policy, defined as a mapping from patient states to treatment decisions, that maximizes the expected cumulative QALY over the decision horizon.

#### 3.1.1 Synthetic data generation

We created a synthetic cohort of *N* = 500 patients observed over *T* = 40 quarterly visits using a Monte Carlo simulation. Ages were sampled uniformly from integers 71 to 95 as a pre-specified simulation parameter. This uniform distribution was selected to ensure adequate representation and algorithmic stress-testing across the entire older adult spectrum, rather than fitting to a specific empirical cohort distribution. At baseline, participants were probabilistically assigned to the frail (70%) or fit (30%) states. This parameterization serves as a cohort-level modeling assumption and reflects the study focus on vulnerable or frail older patients of the target population. Initial dosing incorporated CGA as a part of treatment decision making: fit patients started on full dose, whereas frail patients underwent a Bernoulli trial with *p* = 0.5 to determine assignment to a full or reduced dose. This randomized controlled design enables the comparison of the two treatment strategies (lower initial dose vs. full initial dose) by minimizing potential confounders. At each visit, several time-varying variables were updated, including the clinical test result, frailty status, treatment dose, and QoL. The introduced rules for synthetic data generation are summarized in the Supplementary Table S1.

All time-varying variables in the synthetic dataset were generated using predefined stochastic or rule-based procedures. The clinical-profile variable was generated through a Bernoulli process whose probability depended on the previous clinical profile, treatment dose, and cumulative exposure. Frailty status was updated using age-dependent transition probabilities modified by dose-related toxicity and clinical profile. Treatment dose was initialized according to the study design and subsequently updated using rule-based dose-modification logic, including probabilistic dose reduction under an unfavorable clinical profile. QoL was sampled from frailty-specific uniform distributions, with additional age-related penalties and Gaussian noise. The rules were designed to reflect clinically plausible relationships between frailty status, treatment tolerability, toxicity-related dose adaptation, and QoL in older breast cancer patients, consistent with the clinical rationale of frailty-informed dose optimization in this population (Valachis et al., 2024). We emphasize that these variables should be interpreted as synthetic probabilistic representations of clinical and geriatric trajectories, rather than actual (observed) clinical measurements, and therefore the results of the current application scenario are not intended to directly guide treatment decisions. The Python source code used for synthetic data generation is publicly available.^1^

#### 3.1.2 Clinical test

At each visit, clinical status was simulated as a binary composite indicator (0: unfavorable; 1: favorable). This represented a holistic summary of the patient's condition rather than a specific biochemical or molecular measurement. The probability of a favorable result at the next visit was modeled as a rule-based function of the previous test result, current treatment dose, and cumulative treatment exposure. Specifically, the probability was initialized according to the previous clinical status, adjusted by dose-specific modifiers and cumulative exposure effects, and then used in a Bernoulli trial to determine the next test outcome.

#### 3.1.3 Frailty transitions

For every visit, frailty status was updated using age-dependent transition probabilities modified by treatment dose and clinical profile. Full-dose treatment was modeled as an implicit toxicity-related modifier increasing the probability of transition from fit to frail, whereas reduced dose and favorable clinical profile increased the probability of transition from frail to fit. The parameter values used for these synthetic frailty transitions are provided in Supplementary Table S2.

#### 3.1.4 Treatment dose

Mimicking clinical practice, the dose could be reduced or held in response to the previous visit's clinical assessment. If the clinical_test indicated a poor health state, reduced_dose was selected with probability 0.20 for the current visit. Otherwise, the dose level was carried forward unchanged.

#### 3.1.5 Quality of life

QoL was assumed to depend on frailty status, age, and received therapy (dose). It was sampled from uniform distributions with bounds of [0.6, 0.9] for fit patients and [0.4, 0.7] for frail patients. These intervals represent the range of possible QoL values rather than transition probabilities. An age penalty of 0.015 was subtracted for each year above 75, supplemented by Gaussian noise $N\left(0,0.03^{2}\right)$ to reflect inter-individual variability and stochastic fluctuations in clinical status. The resulting value was clipped to [0, 1] and rounded to two decimals before storage. We note that empirical calibration using real longitudinal QoL data will be required before any clinical interpretation or treatment-assessment.

**Figure 4:**
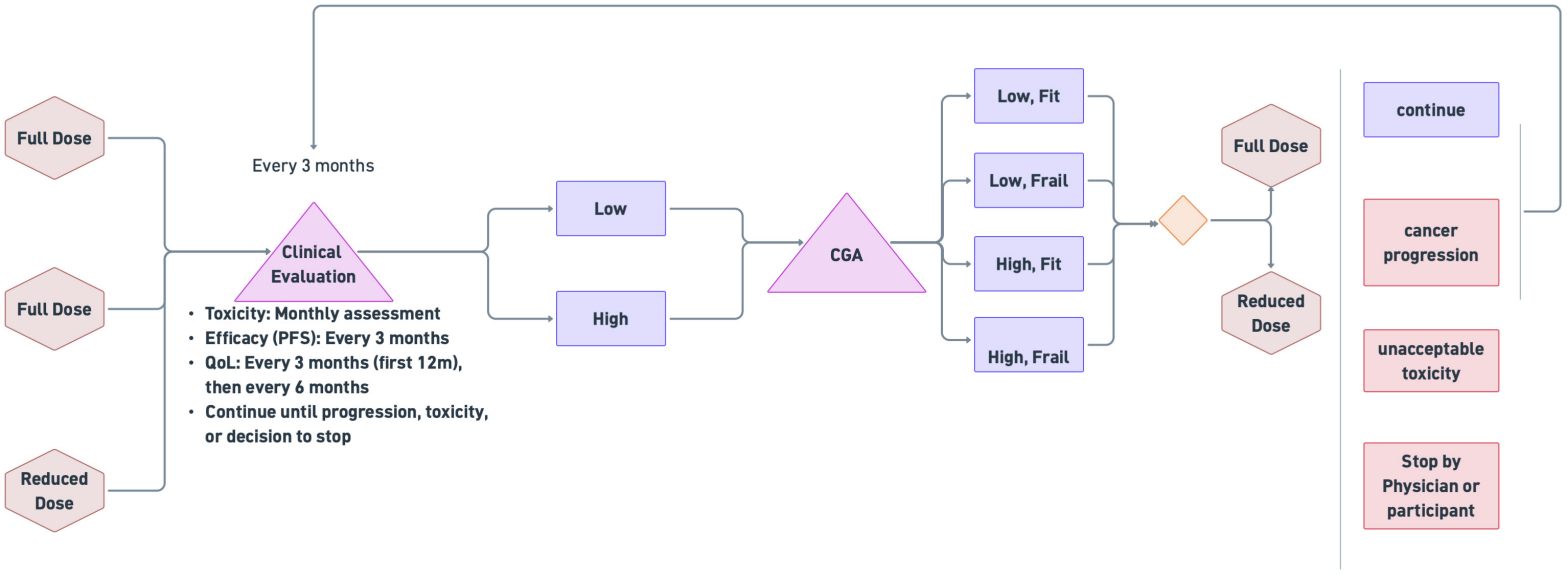
Patients are monitored longitudinally with scheduled clinical evaluations (toxicity, efficacy, quality of life) and repeated geriatric assessment. Based on updated frailty status and clinical outcomes, patients transition into new states (e.g., low/high risk, fit/frail). At each decision point, treatment may be maintained, reduced, escalated, or discontinued. Outcomes such as progression, unacceptable toxicity, or discontinuation are explicitly represented. This adaptive loop provides the basis for the digital twin's MDP framework.

The follow-up phase integrated repeated CGA evaluations every six months, in conjunction with the existing clinical evaluations (Figure 4). In a real clinical implementation, changes in frailty are measured through structured CGA domains, including functional, cognitive, comorbidity, nutritional, psychosocial, and polypharmacy (Sattar et al., 2014; Rockwood and Mitnitski, 2007). For the development of the synthetic dataset, we utilized only the final composite variable–the frailty score–as this metric (along with the clinical test) serves as the primary determinant driving treatment decisions. At each follow-up, treatment decisions (e.g., whether to maintain, escalate, reduce, or discontinue the dose) could thus be informed by both updated CGA findings and clinical test results.

### 3.2 Markov decision process framework

Second, we developed a computational framework based on an MDP to model patient responses under alternative treatment strategies. This framework enabled the estimation of an optimal policy, providing a principled basis for evaluating and guiding therapeutic decision-making. The MDP recommends the dosing action that maximizes expected cumulative QALY. To solve the MDP, we employed value iteration, a dynamic programming algorithm that iteratively updates the expected utility of each state using the Bellman optimality equation until convergence. The resulting value function is then used to derive the optimal policy, defined as the treatment action that maximizes expected cumulative QALY for each patient state (Puterman, 2014). States encode frailty status (Fit/Frail), recent clinical test outcome (favorable/unfavorable), and dosing level (full/reduced). Actions are allowable dose adjustments, rewards reflect the QALY gained during that period, balancing symptom relief with toxicity burden. Given the current state and observed outcomes (CGA + clinical), the MDP updates the patient state and selects the optimal next action via dynamic programming. This process facilitates a mathematically rigorous sequential decision framework, explicitly trading off short-term toxicity against long-term benefit (McCullum et al., 2024; Singh et al., 2025, 2024; Imani et al., 2020).

The MDP estimation used 15,798 visit-level records from the synthetic cohort, yielding 15,298 observed consecutive state transitions. The state space consisted of eight states defined by frailty status, clinical profile, and dose level, and the action space consisted of two dosing actions: reduced dose and full dose. Transition probabilities *P*(*s*_*t*+1_∣*s*_*t*_, *a*_*t*_) were estimated empirically from observed state–action–next-state triplets in the synthetic trajectories, with smoothing applied to account for sparse state–action combinations. Rewards were expressed as QALYs per 3-month cycle, computed from QoL values over a quarterly time step. The final estimated rewards ranged from 0.046 to 0.122 QALYs per visit. Value iteration was performed using a quarterly discount factor corresponding approximately to a 3% annual discount rate (γ≈0.992 per 3-month cycle) to identify the policy that maximized expected cumulative QALYs. The MDP components, estimated reward matrix, final MDP-derived optimal policy, and dominant observed transition probabilities are provided in the Supplementary Tables S3–S6.

## 4 Results

**Figure 5:**
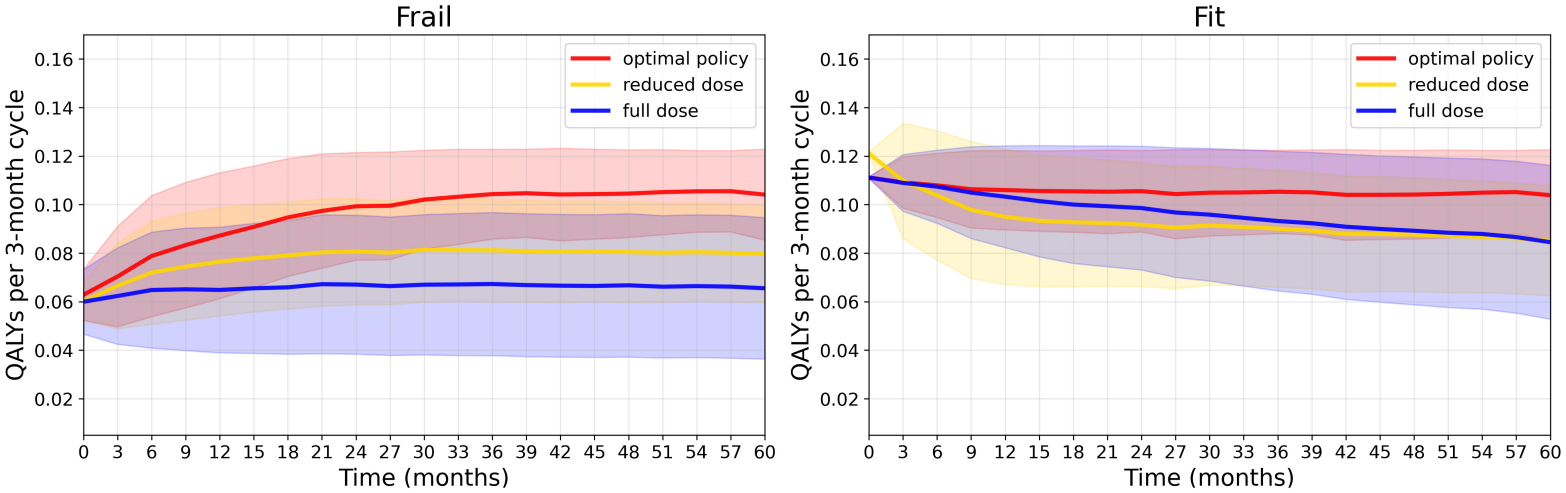
QALY trajectory analysis comparing the optimal MDP-derived dosing policy against alternative treatment strategies for frail patients **(left)** and fit patients **(right)**. Shaded regions represent 95% confidence intervals from Monte Carlo simulations. The optimal adaptive policy maintains higher per-cycle QALY gains than fixed dosing in both frail and fit cohorts, illustrating the benefit of MDP-based dose individualization.

Quality-adjusted life-year trajectories were examined for the initial frail and fit groups (Figure 5). The optimal policy calculated from the generated dataset indicates that fit patients should sustain their current dosage (full or reduced) until they exhibit signs of frailty, while frail patients should alternate between dosage levels, avoiding prolonged periods of continuous full-dose treatment. Under the MDP-optimal policy, the QALY trajectories were compared against fixed full-dose and fixed reduced-dose strategies (Figure 5). Among frail patients (full or reduced dose at baseline), the QALY metric rises steadily under the optimal policy and separates early across treatment groups, indicating that moderation of dose improves tolerability. The QALY for fit patients starts higher at baseline and gradually declines due to cumulative toxicity over time, but the optimal policy mitigates this decline via selective dose reductions. Across the combined cohort (not shown in Figure 5), the optimal policy maintains the highest QALY trajectory over time, demonstrating robust advantages from state-aware dosing. These simulated results demonstrate how the integration of geriatric assessment data within an adaptive, state-transition decision framework could guide individualized dosing decisions. This use case underscores the broader potential of digital-twin technologies to align therapeutic intensity with patient resilience, improving QoL and overall outcomes in geriatric oncology.

## 5 Discussion

Aging has a significant impact on cancer, particularly in determining treatment strategies. Recognizing the need to better understand the unique biology of older cancer patients, in this paper, we present research works that utilized mechanistic models or data-driven techniques in the study of cancer and aging. AI/ML can improve mechanistic models by capturing complex, interconnected biological processes across different scales. These technologies can then be used to create dynamic, multiscale DTs for older breast cancer patients through integration of the individual's medical data along with insights from similar cases within the population. Designed as a virtual replica, the DTs can track the patients' historical health data, monitor their current condition, and predict future outcomes. By dynamically exploring potential treatment pathways, the most effective approach tailored to the specific needs of the older cancer patient can be identified.

A functional DT architecture in geriatric oncology would require integration across multiple layers (Pellegrino et al., 2025). Several types of integration and interfaces are possible, such as on the organ-system interaction level (Sharma and Kaur, 2025), data level (original, simulated), or physiological level. For example, previous work (Hemdan and Sayed, 2025) mirrors the layered data-processing pipelines in current healthcare DT research by defining interfaces between the data layer (that includes population statistics and individual patient biomarkers) and the virtual layer (that contains the modeling and digital representations). Others (Khoshfekr Rudsari et al., 2025) analyze the implementation of DTs across different physiological levels–from cellular to whole-body systems. While these works are not focused on geriatric oncology, the conceptual architectures of healthcare digital twins share fundamental principles across domains. Importantly, such system interfaces should support bidirectional feedback, so that observed treatment responses, adverse events, frailty changes, and QoL trajectories can be incorporated to update patient-state estimates and refine future treatment recommendations (Olawade et al., 2026).

As a practical example, we demonstrated how probabilistic models can leverage data trajectories to estimate state-transition probabilities and cumulative long-term QALY gains in older breast cancer patients. An advanced computational technique, value iteration, was applied to identify optimal dosing strategies. The illustrated MDP approach exemplifies how learning algorithms can significantly enhance personalized treatment strategies. The dual-assessment monitoring changes in frailty and functional status, along with the standard clinical evaluation, demonstrated the potential to support an adaptive treatment strategy over time, particularly in the frail subgroup, with the capacity to improve both tolerability and overall clinical outcomes.

While the demonstrated case study focuses on systematic geriatric assessment, the MDP-based decision framework could be extended to a multiscale setting by incorporating biologically informed state variables derived from mechanistic models. For example, pathway- or signaling-based tools (e.g., CellDesigner and Cell Collective) could be used to process high-dimensional molecular data and generate mechanistic biomarkers, such as pathway activation scores or biological state scores. More comprehensive physiologically based models, including QSP, physiologically based pharmacokinetic/pharmacodynamic, or QSP for immuno-oncology frameworks (Sové et al., 2020; Wang et al., 2024), could further provide biologically grounded estimates of tumor response, drug exposure, immune activation, pathway activity, or toxicity risk. AI/ML methods could support this integration by calibrating mechanistic model parameters from multimodal and multi-omics data, identifying patient subgroups, estimating uncertainty, and developing computationally efficient surrogate models. These mechanistic outputs could then be incorporated into the MDP as state variables or transition estimates, allowing future DT implementations to optimize sequential treatment decisions using both clinical/geriatric trajectories and patient-specific representations of tumor biology, host physiology, and drug action.

The feasibility of clinically useful DTs depends critically on the completeness, granularity, and longitudinal quality of the available data, particularly when the goal is to predict treatment response rather than only to support clinical-state monitoring or dose-adjustment decisions. Omics-based approaches have already been applied in selected clinical and translational oncology contexts, including gene-expression assays that support chemotherapy or endocrine-therapy-related decisions in breast cancer (Paik et al., 2004; Noordhoek et al., 2021). However, these applications differ from fully individualized, longitudinal DTs that dynamically integrate clinical, geriatric, molecular, imaging, treatment-exposure, toxicity, and patient-reported outcome data over time. This distinction is also relevant to the present MDP implementation: in its current form, the model uses discretized patient states and transition probabilities estimated from cohort trajectories. While it provides a robust framework for exploring group-level policy behavior and long-term QALY trade-offs, individual-level DTs require more granular approaches—such as microsimulation—which utilize a patient's specific profile to calculate conditional transition probabilities via regression models (Çağlayan et al., 2018).

In the present application example, which is motivated by a pragmatic clinical trial (the IMPORTANT study), comprehensive multi-omics data were outside the scope of the modeled dataset. Therefore, the current MDP should not be interpreted as a molecular treatment-response predictor. Rather, it illustrates how a probabilistic decision-support layer could be constructed and later coupled with richer multimodal and multi-omics data, including mechanistic or QSP/PBPK-derived biological state variables, when such data become available. Future implementations should prioritize high-fidelity longitudinal datasets and, where feasible, multi-omics integration to improve biologically grounded patient-state representations and treatment-response predictions.

It should be noted, however, that the transition from the proof-of-concept case study presented here to a fully integrated clinical DT faces substantial challenges. As highlighted by the TRIAD framework (Li et al., 2025), for a DT to be useful in geriatric oncology, it must be part of an integrated adaptive deployment strategy. This means the model should not only predict outcomes but also adapt to the shifting health status of an elderly patient, while maintaining trustworthy governance to ensure that automated dosing suggestions are transparent and ethically sound for vulnerable populations. Moreover, the integration of longitudinal and multimodal data—encompassing genomic, clinical, and QoL measures requires rigorous data standardization. Without it, the flexible AI approaches, discussed in this survey, risk becoming “black boxes” that lack the interoperability necessary for clinical use. As part of our future work, we intend to calibrate and validate the MDP framework using longitudinal real-world data from the IMPORTANT project. In this setting, frailty status, CGA domains, clinical outcomes, toxicity, and QoL measures will be derived from actual patient assessments rather than simulations, allowing the transition probabilities, reward function, and resulting treatment policies to be empirically evaluated and iteratively refined.

Beyond this application, embedding age-related metrics within oncology-focused DTs would allow models to represent not only tumor dynamics but also the broader aging trajectory of the patients (Mandelblatt et al., 2021). Evidence indicates that “hallmarks” of aging (e.g., genomic instability, telomere attrition, epigenetic alterations) overlap with cancer pathways, influencing both carcinogenesis and tumor progression (Montégut et al., 2024). To enable earlier risk stratification and preventive interventions, digital twins should incorporate functional (mobility, cognition), physiological (metabolic state), and molecular (omics-based) markers of aging alongside cancer-specific data, such as pathological tumor profiles (stage and grade), longitudinal treatment response, and trajectories of disease progression. This integration is particularly critical in geriatric oncology, where treatment tolerance, toxicity, and long-term outcomes depend on the interplay between cancer progression and age-related decline. By uniting cancer modeling with aging trajectories, holistic digital twins could better guide therapy selection, balancing oncological efficacy with healthspan preservation and quality of life in older populations (Battisti and Dotan, 2020).

## Data Availability

The data analyzed in this study were synthetically generated. The Python source code used to generate the synthetic data is publicly available at: https://github.com/PanagiotisKarampelesis/synthetic-data-generation-geriatric-oncology.

## Footnotes

1
https://github.com/PanagiotisKarampelesis/synthetic-data-generation-geriatric-oncology.git

## References

[B1] Abdollahi H. Yousefirizi F. Shiri I. Brosch-Lenz J. Mollaheydar E. Fele-Paranj A. . (2024). Theranostic digital twins: concept, framework and roadmap towards personalized radiopharmaceutical therapies. Theranostics 14:3404. doi: 10.7150/thno.93973 38948052 PMC11209714

[B2] Aghamiri S. S. Singh V. Naldi A. Helikar T. Soliman S. Niarakis A. (2020). Automated inference of boolean models from molecular interaction maps using CASQ. Bioinformatics 36, 4473–4482. doi: 10.1093/bioinformatics/btaa484 32403123 PMC7575051

[B3] Arjmand B. Hamidpour S. K. Tayanloo-Beik A. Goodarzi P. Aghayan H. R. Adibi H. . (2022). Machine learning: a new prospect in multi-omics data analysis of cancer. Front. Genet. 13:824451. doi: 10.3389/fgene.2022.824451 35154283 PMC8829119

[B4] Battisti N. M. L. Dotan E. (2020). “Integrating geriatric oncology into clinical pathways and guidelines,” in Geriatric Oncology (Cham: Springer), 23–35.

[B5] Bennett C. C. Hauser K. (2013). Artificial intelligence framework for simulating clinical decision-making: a Markov decision process approach. Artif. Intell. Med. 57, 9–19. doi: 10.1016/j.artmed.2012.12.003 23287490

[B6] Berben L. Floris G. Wildiers H. Hatse S. (2021). Cancer and aging: two tightly interconnected biological processes. Cancers 13:1400. doi: 10.3390/cancers13061400 33808654 PMC8003441

[B7] Boehm K. M. Khosravi P. Vanguri R. Gao J. Shah S. P. (2022). Harnessing multimodal data integration to advance precision oncology. Nat. Rev. Cancer 22, 114–126. doi: 10.1038/s41568-021-00408-3 34663944 PMC8810682

[B8] Bozcuk H. S. Sert L. Kaplan M. A. Tatlı A. M. . (2025). Enhancing treatment decisions for advanced non-small cell lung cancer with epidermal growth factor receptor mutations: a reinforcement learning approach. Cancers 17:233. doi: 10.3390/cancers17020233 39858018 PMC11763509

[B9] Çağlayan Ç. Terawaki H. Chen Q. Rai A. Ayer T. Flowers C. R. (2018). Microsimulation modeling in oncology. JCO Clini. Cancer Inform. 2, 1–11. doi: 10.1200/CCI.17.00029 PMC6386553 30652551

[B10] Cassidy R. Singh N. S. Schiratti P.-R. Semwanga A. Binyaruka P. Sachingongu N. . (2019). Mathematical modelling for health systems research: a systematic review of system dynamics and agent-based models. BMC Health Serv. Res. 19:845. doi: 10.1186/s12913-019-4627-7 31739783 PMC6862817

[B11] Chan W.-L. Lau S. K.-W. Mak A. Yau C.-M. Fung C.-F. Hou H. L.-Y. . (2025). Prediction models for severe treatment-related toxicities in older adults with cancer: a systematic review. Age and Ageing 54:afaf095. doi: 10.1093/ageing/afaf095 40253686

[B12] Chandler Y. Schechter C. B. Jayasekera J. Near A. O'Neill S. C. Isaacs C. . (2018). Cost effectiveness of gene expression profile testing in community practice. J. Clini. Oncol. 36, 554–562. doi: 10.1200/JCO.2017.74.5034 PMC5815401 29309250

[B13] Chodera J. D. Noé F. (2014). Markov state models of biomolecular conformational dynamics. Curr. Opin. Struct. Biol. 25, 135–144. doi: 10.1016/j.sbi.2014.04.002 24836551 PMC4124001

[B14] Chu W.-M. Tsan Y.-T. Chen P.-Y. Chen C.-Y. Hao M.-L. Chan W.-C. . (2023). A model for predicting physical function upon discharge of hospitalized older adults in taiwan–a machine learning approach based on both electronic health records and comprehensive geriatric assessment. Front. Med. 10:1160013. doi: 10.3389/fmed.2023.1160013 37547611 PMC10400801

[B15] Clermont G. Vodovotz Y. Rubin J. (2007). Equation-based models of dynamic biological systems. Endothelial Biomed. 2007, 1780–1785. doi: 10.1017/CBO9780511546198.192 PMC6698907 19056027

[B16] Deng J. Hartung T. Capobianco E. Chen J. Y. Emmert-Streib F. (2022). Artificial intelligence for precision medicine. Front. Artif . Intellig. 4:834645. doi: 10.3389/978-2-88974-488-6 PMC8814648 35128393

[B17] Eastman B. Przedborski M. Kohandel M. (2021). Reinforcement learning derived chemotherapeutic schedules for robust patient-specific therapy. Sci. Rep. 11:17882. doi: 10.1038/s41598-021-97028-6 34504141 PMC8429726

[B18] Fabris F. Magalh aes J. P.d Freitas A. A. (2017). A review of supervised machine learning applied to ageing research. Biogerontology 18, 171–188. doi: 10.1007/s10522-017-9683-y 28265788 PMC5350215

[B19] Funahashi A. Morohashi M. Kitano H. Tanimura N. (2003). Celldesigner: a process diagram editor for gene regulatory and biochemical networks. Biosilico 1. doi: 10.1016/S1478-5382(03)02370-9

[B20] Ge H. Qian M. (2009). Boolean network approach to negative feedback loops of the p53 pathways: synchronized dynamics and stochastic limit cycles. J. Comput. Biol. 16, 119–132. doi: 10.1089/cmb.2007.0181 19119996

[B21] George J. T. Levine H. (2018). Stochastic modeling of tumor progression and immune evasion. J. Theor. Biol. 458, 148–155. doi: 10.1016/j.jtbi.2018.09.012 30218648 PMC6225903

[B22] Ghislain M. Martin F. Dausort M. Dasnoy-Sumell D. Barragan Montero A. M. Macq B. (2025). Optimal fractionation scheduling for radiotherapy treatments with reinforcement learning, tumor growth modeling and outcome modeling. Biomedicines 13:1367. doi: 10.3390/biomedicines13061367 40564086 PMC12190542

[B23] Golzy M. Rosen G. H. Kruse R. L. Hooshmand K. Mehr D. R. Murray K. S. (2023). Holistic assessment of quality of life predicts survival in older patients with bladder cancer. Urology 174, 141–149. doi: 10.1016/j.urology.2022.12.036 36669573

[B24] Gonzalez A. G. Naldi A. Snchez L. Thieffry D. Chaouiya C. (2006). GINSIM: a software suite for the qualitative modelling, simulation and analysis of regulatory networks. Biosystems 84, 91–100. doi: 10.1016/j.biosystems.2005.10.003 16434137

[B25] Gudkov A. V. Komarova E. A. (2016). p53 and the carcinogenicity of chronic inflammation. Cold Spring Harb. Perspect. Med. 6:a026161. doi: 10.1101/cshperspect.a026161 27549311 PMC5088512

[B26] Guerard E. J. Deal A. M. Chang Y. Williams G. R. Nyrop K. A. Pergolotti M. . (2017). Frailty index developed from a cancer-specific geriatric assessment and the association with mortality among older adults with cancer. J. Nat. Comprehen. Cancer Netw. 15, 894–902. doi: 10.6004/jnccn.2017.0122 28687577

[B27] Helikar T. Kowal B. McClenathan S. Bruckner M. Rowley T. Madrahimov A. . (2012). The cell collective: toward an open and collaborative approach to systems biology. BMC Syst. Biol. 6:96. doi: 10.1186/1752-0509-6-96 22871178 PMC3443426

[B28] Hemdan E. E.-D. Sayed A. (2025). Smart and secure healthcare with digital twins: a deep dive into blockchain, federated learning, and future innovations. Algorithms 18:7. doi: 10.3390/a18070401

[B29] Heudel P. Ahmed M. Renard F. Attye A. (2025). Leveraging digital twins for stratification of patients with breast cancer and treatment optimization in geriatric oncology: Multivariate clustering analysis. JMIR Cancer 11:e64000. doi: 10.2196/64000 40408774 PMC12124816

[B30] Hoops S. Sahle S. Gauges R. Lee C. Pahle J. Simus N. . (2006). Copasi'a complex pathway simulator. Bioinformatics 22, 3067–3074. doi: 10.1093/bioinformatics/btl485 17032683

[B31] Hucka M. Bergmann F. T. Hoops S. Keating S. M. Sahle S. Schaff J. C. . (2015). The systems biology markup language (SBML): Language specification for level 3 version 1 core. J. Integr. Bioinform. 12, 382–549. doi: 10.1515/jib-2015-266 26528564 PMC5451324

[B32] Hurria A. Togawa K. Mohile S. G. Owusu C. Klepin H. D. Gross C. P. . (2011). Predicting chemotherapy toxicity in older adults with cancer: a prospective multicenter study. J. Clini. Oncol. 29, 3457–3465. doi: 10.1200/JCO.2011.34.7625 21810685 PMC3624700

[B33] Imani F. Qiu Z. Yang H. (2020). “Markov decision process modeling for multi-stage optimization of intervention and treatment strategies in breast cancer,” in 2020 42nd Annual International Conference of the IEEE Engineering in Medicine & Biology Society (EMBC) (Montreal, QC: IEEE), 5394–5397. 10.1109/EMBC44109.2020.9175905 33019200

[B34] IMPORTANT Project (2023). Implementing Geriatric Assessment for Dose Optimization of CDK 4/6-Inhibitors in Older Breast Cancer Patients. Available online at: https://important-project.com/ (Accessed May 1, 2023–April 30, 2028).

[B35] Jordan M. I. Mitchell T. M. (2015). Machine learning: trends, perspectives, and prospects. Science 349, 255–260. doi: 10.1126/science.aaa8415 26185243

[B36] Kanehisa M. Furumichi M. Sato Y. Matsuura Y. Ishiguro-Watanabe M. (2024). KEGG: biological systems database as a model of the real world. Nucleic Acids Res. 53, D672–D677. doi: 10.1093/nar/gkae909 39417505 PMC11701520

[B37] Karaman I. Sebin B. (2025). From data-driven cities to data-driven tumors: dynamic digital twins for adaptive oncology. Front. Artif. Intellig. 8:1624877. doi: 10.3389/frai.2025.1624877 40785836 PMC12331622

[B38] Katsoulakis E. Wang Q. Wu H. Shahriyari L. Fletcher R. Liu J. . (2024). Digital twins for health: a scoping review. NPJ Digit. Med. 7:77. doi: 10.1038/s41746-024-01073-0 38519626 PMC10960047

[B39] Khoshfekr Rudsari H. Tseng B. Zhu H. Song L. Gu C. Roy A. . (2025). Digital twins in healthcare: a comprehensive review and future directions. Front. Digital Health 7:1633539. doi: 10.3389/fdgth.2025.1633539 41341463 PMC12671388

[B40] Lagergren J. H. Nardini J. T. Baker R. E. Simpson M. J. Flores K. B. (2020). Biologically-informed neural networks guide mechanistic modeling from sparse experimental data. PLoS Comput. Biol. 16:e1008462. doi: 10.1371/journal.pcbi.1008462 33259472 PMC7732115

[B41] Lefèvre A. Parra-Guillen Z. P. Trocóniz I. F. Boetsch C. Frances N. (2025). Mechanistic PKPD modeling to describe cytokine release associated with cd3 t-cell engager therapies. Front. Immunol. 15:1463915. doi: 10.3389/fimmu.2024.1463915 39896804 PMC11782561

[B42] Li J. Zhou Z.-C. Wang Z.-C. Lv H. (2025). Prioritizing human-ai collaboration in healthcare: the triad framework for trustworthy governance, real-world, and integrated adaptive deployment. Military Med. Res. 12:97. doi: 10.1186/s40779-026-00684-w 41810150 PMC12888660

[B43] Lopez C. Tucker S. Salameh T. Tucker C. (2018). An unsupervised machine learning method for discovering patient clusters based on genetic signatures. J. Biomed. Inform. 85:30–39. doi: 10.1016/j.jbi.2018.07.004 30016722 PMC6621561

[B44] Lorenzo G. Ahmed S. R. Hormuth D. A. Vaughn B. Kalpathy-Cramer J. Solorio L. . (2024). Patient-specific, mechanistic models of tumor growth incorporating artificial intelligence and big data. Annu. Rev. Biomed. Eng. 26, 529–560. doi: 10.1146/annurev-bioeng-081623-025834 38594947

[B45] Lyu M. Yi S. Li C. Xie Y. Liu Y. Xu Z. . (2025). Multimodal prediction based on ultrasound for response to neoadjuvant chemotherapy in triple negative breast cancer. NPJ Precisi. Oncol. 9:259. doi: 10.1038/s41698-025-01057-7 40715366 PMC12297240

[B46] Majumder D. (2023). Identification of residual disease followed by trade-off analysis between drug optimisation, MRD and sustenance of normal haematopoiesis under maintenance chemotherapy in childhood all. Int. J. Model. Identif. Cont. 42, 360–374. doi: 10.1504/IJMIC.2023.131206

[B47] Mandelblatt J. S. Ahles T. A. Lippman M. E. Isaacs C. (2021). Applying a life course biological age framework to improving the care of individuals with adult cancers. JAMA Oncology 7, 1692–1699. doi: 10.1001/jamaoncol.2021.1160 34351358 PMC8602673

[B48] Martinez-Jimenez C. P. Eling N. Chen H.-C. Vallejos C. A. Kolodziejczyk A. A. Connor F. . (2017). Aging increases cell-to-cell transcriptional variability upon immune stimulation. Science 355, 1433–1436. doi: 10.1126/science.aah4115 28360329 PMC5405862

[B49] Mascheroni P. Savvopoulos S. Alfonso J. C. L. Meyer-Hermann M. Hatzikirou H. (2021). Improving personalized tumor growth predictions using a bayesian combination of mechanistic modeling and machine learning. Commun. Med. 1:19. doi: 10.1038/s43856-021-00020-4 35602187 PMC9053281

[B50] Mashayekhi H. Nazari M. Jafarinejad F. Meskin N. (2024). Deep reinforcement learning-based control of chemo-drug dose in cancer treatment. Comput. Methods Programs Biomed. 243:107884. doi: 10.1016/j.cmpb.2023.107884 37948911

[B51] McCullum L. B. Karagoz A. Dede C. Garcia R. Nosrat F. Hemmati M. . (2024). Markov models for clinical decision-making in radiation oncology: a systematic review. J. Med. Imaging Radiat. Oncol. 68, 610–623. doi: 10.1111/1754-9485.13656 38766899 PMC11576491

[B52] Meeson K. E. Schwartz J.-M. (2024). Constraint-based modelling predicts metabolic signatures of low and high-grade serous ovarian cancer. NPJ Syst. Biol. Appl. 10:96. doi: 10.1038/s41540-024-00418-5 39181893 PMC11344801

[B53] Metzcar J. Jutzeler C. R. Macklin P. Köhn-Luque A. Brüningk S. C. (2024). A review of mechanistic learning in mathematical oncology. Front. Immunol. 15:1363144. doi: 10.3389/fimmu.2024.1363144 38533513 PMC10963621

[B54] Milacic M. Beavers D. Conley P. Gong C. Gillespie M. Griss J. . (2023). The reactome pathway knowledgebase 2024. Nucleic Acids Res. 52, D672–D678. doi: 10.1093/nar/gkad1025 37941124 PMC10767911

[B55] Montégut L. Lpez-Otn C. Kroemer G. (2024). Aging and cancer. Mol. Cancer 23:106. doi: 10.1186/s12943-024-02020-z 38760832 PMC11102267

[B56] Naldi A. (2018). Biolqm: a java toolkit for the manipulation and conversion of logical qualitative models of biological networks. Front. Physiol. 9:1605. doi: 10.3389/fphys.2018.01605 30510517 PMC6254088

[B57] Naldi A. Monteiro P. T. Mssel C. the Consortium for Logical Models Tools, Kestler, H. A. Thieffry D. Xenarios I. . (2015). Cooperative development of logical modelling standards and tools with colomoto. Bioinformatics 31, 1154–1159. doi: 10.1093/bioinformatics/btv013 25619997

[B58] Noordhoek I. Treuner K. Putter H. Zhang Y. Wong J. Meershoek-Klein Kranenbarg E. . (2021). Breast cancer index predicts extended endocrine benefit to individualize selection of patients with HR+ early-stage breast cancer for 10 years of endocrine therapy. Clinical Cancer Res. 27, 311–319. doi: 10.1158/1078-0432.CCR-20-2737 33109739

[B59] Olawade D. B. Oisakede E. O. Bello O. J. Analikwu C. C. Egbon E. Ojo A. (2026). Digital twins in oncology: from predictive modelling to personalised treatment strategies. Criti. Rev. Oncol./Hematol. 2026:105171. doi: 10.1016/j.critrevonc.2026.105171 41621635

[B60] Othman N. A. Abdel-Fattah M. A. Ali A. T. (2023). A hybrid deep learning framework with decision-level fusion for breast cancer survival prediction. Big Data Cognit. Comput. 7:50. doi: 10.3390/bdcc7010050

[B61] Oughtred R. Rust J. Chang C. Breitkreutz B.-J. Stark C. Willems A. . (2021). The biogrid database: a comprehensive biomedical resource of curated protein, genetic, and chemical interactions. Protein Sci. 30, 187–200. doi: 10.1002/pro.3978 33070389 PMC7737760

[B62] Paik S. Shak S. Tang G. Kim C. Baker J. Cronin M. . (2004). A multigene assay to predict recurrence of tamoxifen-treated, node-negative breast cancer. New Engl. J. Med. 351, 2817–2826. doi: 10.1056/NEJMoa041588 15591335

[B63] Parvin N. Joo S. W. Jung J. H. Mandal T. K. (2025). Multimodal ai in biomedicine: Pioneering the future of biomaterials, diagnostics, and personalized healthcare. Nanomaterials 15:895. doi: 10.3390/nano15120895 40559258 PMC12195918

[B64] Pellegrino G. Gervasi M. Angelelli M. Corallo A. (2025). A conceptual framework for digital twin in healthcare: evidence from a systematic meta-review. Inform. Syst. Front. 27, 7–32. doi: 10.1007/s10796-024-10536-4

[B65] Pilleron S. O'Hanlon S. (2023). Digital twins for geriatric oncology: Double trouble or twice as nice? J. Geriatr. Oncol. 14:101524. doi: 10.1016/j.jgo.2023.101524 37208231

[B66] Procopio A. Cesarelli G. Donisi L. Merola A. Amato F. Cosentino C. (2023). Combined mechanistic modeling and machine-learning approaches in systems biology-a systematic literature review. Comput. Methods Programs Biomed. 240:107681. doi: 10.1016/j.cmpb.2023.107681 37385142

[B67] Puterman M. L. (2014). Markov Decision Processes: Discrete Stochastic Dynamic Programming. Hoboken, NJ: John Wiley & Sons.

[B68] Qian L. Wang Z. Paine M. F. Chan E. C. Y. Zhou Z. (2024). Application of physiologically-based pharmacokinetic modeling to inform dosing decisions for geriatric patients. CPT: Pharmacomet. Syst. Pharmacol. 13:2031. doi: 10.1002/psp4.13241 39291626 PMC11646931

[B69] Rabinovici-Cohen S. Fernández X. M. Grandal Rejo B. Hexter E. Hijano Cubelos O. Pajula J. . (2022). Multimodal prediction of five-year breast cancer recurrence in women who receive neoadjuvant chemotherapy. Cancers 14:3848. doi: 10.3390/cancers14163848 36010844 PMC9405765

[B70] Raissi M. Perdikaris P. Karniadakis G. E. (2019). Physics-informed neural networks: A deep learning framework for solving forward and inverse problems involving nonlinear partial differential equations. J. Comput. Phys. 378:686–707. doi: 10.1016/j.jcp.2018.10.045

[B71] Rockwood K. Mitnitski A. (2007). Frailty in relation to the accumulation of deficits. J. Gerontol. Series A: Biol. Sci. Med. Sci. 62, 722–727. doi: 10.1093/gerona/62.7.722 17634318

[B72] Sattar S. Alibhai S. M. Wildiers H. Puts M. T. (2014). How to implement a geriatric assessment in your clinical practice. Oncologist 19, 1056–1068. doi: 10.1634/theoncologist.2014-0180 25187477 PMC4200997

[B73] Schaefer A. J. Bailey M. D. Shechter S. M. Roberts M. S. (2005). “Modeling medical treatment using markov decision processes,” in Operations Research and Health Care: A Handbook of Methods and Applications (Cham: Springer), 593–612.

[B74] Sena G. R. Lima T. P. F. Mello M. J. G. Thuler L. C. S. Lima J. T. O. (2019). Developing machine learning algorithms for the prediction of early death in elderly cancer patients: usability study. JMIR Cancer 5:e12163. doi: 10.2196/12163 31573896 PMC6787529

[B75] Shannon P. Markiel A. Ozier O. Baliga N. S. Wang J. T. Ramage D. . (2003). Cytoscape: a software environment for integrated models of biomolecular interaction networks. Genome Res. 13, 2498–2504. doi: 10.1101/gr.1239303 14597658 PMC403769

[B76] Sharma H. Kaur S. (2025). Patient-specific digital twins for personalized healthcare: A hybrid AI and simulation-based framework. IEEE Access 13, 143277–143290. doi: 10.1109/ACCESS.2025.3598130

[B77] Shirini D. Schwartz L. H. Dercle L. (2023). Artificial intelligence for aging research in cancer drug development. Aging 15:12699. doi: 10.18632/aging.204914 37980599 PMC10713405

[B78] Shive C. Pandiyan P. (2022). Inflammation, immune senescence, and dysregulated immune regulation in the elderly. Front. Aging 3:840827. doi: 10.3389/fragi.2022.840827 35821823 PMC9261323

[B79] Siebert U. Alagoz O. Bayoumi A. M. Jahn B. Owens D. K. Cohen D. J. . (2012). State-transition modeling: a report of the ispor-smdm modeling good research practices task force-3. Med. Deci. Making 32, 690–700. doi: 10.1177/0272989X12455463 22990084

[B80] Singh P. Singh S. Mishra A. Mishra S. K. (2024). Multimodality treatment planning using the markov decision process: a comprehensive study of applications and challenges. Res. Biomed. Eng. 40, 435–450. doi: 10.1007/s42600-024-00349-4

[B81] Singh S. Sahu C. Singh P. Mishra A. Mishra S. K. Patnaik P. K. (2025). Strategic cancer therapy planning: optimizing treatment and quality of life with markov decision processes. Reports Pract. Oncol. Radiother. 30, 735–748. doi: 10.5603/rpor.108580 41498089 PMC12767983

[B82] Sové R. J. Jafarnejad M. Zhao C. Wang H. Ma H. Popel A. S. (2020). Qsp-io: a quantitative systems pharmacology toolbox for mechanistic multiscale modeling for immuno-oncology applications. CPT: Pharmacomet. Syst. Pharmacol. 9, 484–497. doi: 10.1002/psp4.12546 32618119 PMC7499194

[B83] Stoll G. Viara E. Barillot E. Calzone L. (2012). Continuous time boolean modeling for biological signaling: application of gillespie algorithm. BMC Syst. Biol. 6:116. doi: 10.1186/1752-0509-6-116 22932419 PMC3517402

[B84] Szklarczyk D. Kirsch R. Koutrouli M. Nastou K. Mehryary F. Hachilif R. . (2022). The string database in 2023: protein–protein association networks and functional enrichment analyses for any sequenced genome of interest. Nucleic Acids Res. 51, D638–D646. doi: 10.1093/nar/gkac1000 36370105 PMC9825434

[B85] Tardini E. Zhang X. Canahuate G. Wentzel A. Mohamed A. S. R. Van Dijk L. . (2022). Optimal treatment selection in sequential systemic and locoregional therapy of oropharyngeal squamous carcinomas: Deep q-learning with a patient-physician digital twin dyad. J. Med. Internet Res. 24:e29455. doi: 10.2196/29455 35442211 PMC9069283

[B86] Tong L. Mitchel J. Chatlin K. Wang M. D. (2020). Deep learning based feature-level integration of multi-omics data for breast cancer patients survival analysis. BMC Med. Inform. Decis. Mak. 20:225. doi: 10.1186/s12911-020-01225-8 32933515 PMC7493161

[B87] Vahabi N. Michailidis G. (2022). Unsupervised multi-omics data integration methods: a comprehensive review. Front. Genet. 13:854752. doi: 10.3389/fgene.2022.854752 35391796 PMC8981526

[B88] Valachis A. Biganzoli L. Christopoulou A. Fjermeros K. Fountzila E. Geisler J. . (2024). Implementing geriatric assessment for dose optimization of cdk4/6 inhibitors in older breast cancer patients. Future Oncology 20, 2937–2948. doi: 10.1080/14796694.2024.2413841 39431459 PMC11572072

[B89] Wang H. Arulraj T. Ippolito A. Popel A. S. (2024). From virtual patients to digital twins in immuno-oncology: lessons learned from mechanistic quantitative systems pharmacology modeling. NPJ Digit. Med. 7:189. doi: 10.1038/s41746-024-01188-4 39014005 PMC11252162

[B90] Welsh T. J. Gordon A. L. Gladman J. (2014). Comprehensive geriatric assessment-a guide for the non-specialist. Int. J. Clin. Pract. 68:290. doi: 10.1111/ijcp.12313 24118661 PMC4282277

[B91] Wentzel A. Attia S. Zhang X. Canahuate G. Fuller C. D. Marai G. E. (2024). Ditto: A visual digital twin for interventions and temporal treatment outcomes in head and neck cancer. IEEE Trans. Visualizat. Comp. Graph. 31, 65–75. doi: 10.1109/TVCG.2024.3456160 39255169 PMC11879760

[B92] Wu C. Lorenzo G. Hormuth D. A. Lima E. A. Slavkova K. P. DiCarlo J. C. . (2022). Integrating mechanism-based modeling with biomedical imaging to build practical digital twins for clinical oncology. Biophys. Rev. 3:2. doi: 10.1063/5.0086789 35602761 PMC9119003

[B93] Xu H. Mohamed M. Flannery M. Peppone L. Ramsdale E. Loh K. P. . (2023). An unsupervised machine learning approach to evaluating the association of symptom clusters with adverse outcomes among older adults with advanced cancer: a secondary analysis of a randomized clinical trial. JAMA Network Open 6, e234198–e234198. doi: 10.1001/jamanetworkopen.2023.4198 36947036 PMC10034574

[B94] Xue Q.-L. (2011). The frailty syndrome: definition and natural history. Clin. Geriatr. Med. 27:1. doi: 10.1016/j.cger.2010.08.009 21093718 PMC3028599

