# Supplementary material for: From mechanistic models to artificial intelligence: exploring the potential of digital twins in geriatric oncology
Source: Front Artif Intell. 2026 Jul 23;9:1760912. doi: 10.3389/frai.2026.1760912 (PMC13443076; doi:10.3389/frai.2026.1760912)
Supplement: Supplementary file 1 [file Data_Sheet_1.pdf]

Supplementary Material

Supplementary Table S1. Synthetic data generation assumptions

The synthetic cohort was generated using Monte Carlo simulation. The parameters below summarize the main modeling assumptions used for the proof-of-concept MDP analysis.

**Component**	**Modeling assumption**
Cohort size	$N = 500$ synthetic patients.
Follow-up horizon	$T = 40$ quarterly visits.
Age distribution	Uniformly sampled integer values from 71 to 95 years.
Baseline frailty	Frail: 70%; Fit: 30%.
Initial dose	Fit patients started on full dose; frail patients were assigned to full or reduced dose with probability $p = 0.5$.
Clinical profile	Binary composite variable updated using a rule-based Bernoulli process conditional on previous clinical profile, dose, and cumulative treatment exposure.
Frailty status	Updated using age-dependent transition probabilities modified by dose and clinical profile; see Supplementary Table S2.
Dose update	Dose was updated using rule-based dose-modification logic; reduced dose was selected with probability 0.20 under an unfavorable clinical profile.
Quality of life	QoL was sampled from frailty-specific uniform distributions, adjusted for age, Gaussian noise, and clipped to the range $[0, 1]$.
CGA representation	CGA-derived vulnerability was represented through an aggregated binary frailty state rather than separate CGA-domain scores.
Interpretation	All parameters represent synthetic modeling assumptions for proof-of-concept analysis and are not clinically validated estimates.

Table S1. Summary of the main synthetic data generation assumptions used in the proof-of-concept analysis.

Supplementary Table S2. Frailty transition probabilities

Frailty status was updated at each visit using age-dependent transition probabilities, modified by treatment dose and clinical profile. These probabilities were used as synthetic modeling assumptions to generate longitudinal frailty trajectories and should not be interpreted as clinically validated estimates of frailty progression or recovery.

**Fit → Frail**	**Frail → Fit**
• Age ≤ 75: $p = 0.014$<br>• $75 <$ age ≤ 80: $p = 0.022$<br>• Age > 80: $p = 0.036$<br>• *Dose-related toxicity modifier*: if the patient is receiving full dose, add $+0.03$ to p.	• Age ≤ 75: $p = 0.010$<br>• $75 <$ age ≤ 80: $p = 0.008$<br>• Age > 80: $p = 0.006$<br>• *Recovery modifier*: add $+0.04$ to p if the patient is receiving reduced dose, and add $+0.003$ if the clinical profile is favorable.

Table S2. Age-dependent frailty transition probabilities and dose/clinical-profile modifiers used in the synthetic data generation process. Full dose was used as an implicit toxicity-related modifier increasing the probability of transition from fit to frail, whereas reduced dose and favorable clinical profile increased the probability of recovery from frail to fit.

Supplementary Table S3. MDP components

The MDP was defined by a finite set of patient states, two possible dosing actions, transition probabilities, and QALY-based rewards.

**Component**	**Values used in the MDP**
State variables	Frailty status, clinical profile, dose level
Frailty status	Fit, Frail
Clinical profile	Negative, Positive
Dose level	Reduced dose, Full dose
State space	All 8 combinations of Frailty status, Clinical profile and Dose level.
Actions	Reduced dose; Full dose
Transition model	$P(s_{t+1} \mid s_t, a_t)$ estimated from synthetic state–action–next-state triplets, with smoothing for sparse combinations
Reward	QALYs per 3-month cycle
Reward range	0.046–0.122 QALYs per 3-month cycle
Discount factor	$\gamma \approx 0.992$ per 3-month cycle, corresponding approximately to a 3% annual discount rate
Optimization method	Value iteration

Table S3. MDP components used in the synthetic proof-of-concept analysis. The MDP should be interpreted as a stochastic clinical and geriatric decision-support layer, not as a molecular or mechanistic tumor-biology model.

Supplementary Table S4. Reward matrix

Rewards were expressed as QALYs per 3-month cycle. The table below reports the final estimated reward values for each MDP state and dosing action used in the value-iteration analysis. Rewards were estimated from the synthetic trajectories; sparse state-action combinations were handled using transition-based estimation.

State	Reduced dose	Full dose
Fit_Neg_Red	0.113	0.118
Fit_Neg_Full	0.120	0.111
Fit_Pos_Red	0.115	0.105
Fit_Pos_Full	0.122	0.112
Frail_Neg_Red	0.069	0.071
Frail_Neg_Full	0.051	0.047
Frail_Pos_Red	0.067	0.075
Frail_Pos_Full	0.053	0.046

Table S4. Reward matrix used in the MDP analysis. Rewards are expressed as QALYs per 3-month cycle for each state-action pair. The final reward range was 0.046–0.122 QALYs per 3-month cycle. These values were estimated from the synthetic trajectories and should be interpreted as proof-of-concept reward assumptions rather than clinically validated utility estimates.

Supplementary Table S5. MDP-derived optimal policy

The optimal policy maps each MDP state to the dosing action that maximizes expected cumulative QALYs under the synthetic proof-of-concept analysis. The actions below should be interpreted as model-derived outputs from the simulation, not as clinical treatment recommendations.

MDP state	Optimal action
Fit_Neg_Red	Reduced dose
Fit_Neg_Full	Full dose
Fit_Pos_Red	Reduced dose
Fit_Pos_Full	Full dose
Frail_Neg_Red	Full dose
Frail_Neg_Full	Reduced dose
Frail_Pos_Red	Full dose
Frail_Pos_Full	Reduced dose

Table S5. Optimal policy derived by value iteration in the synthetic MDP analysis. The policy corresponds to the value-iteration output `[0 1 0 1 1 0 1 0]`, where action 0 denotes reduced dose and action 1 denotes full dose.

Supplementary Table S6. Transition probability summary

Transition probabilities were estimated from consecutive synthetic patient visits using observed state–action–next-state triplets. The table reports the dominant next-state probabilities for state-action combinations with sufficient observations. The count n denotes the total number of observed transitions for the corresponding current state and action.

**Current state**	**Action**	**Observed transitions** (n)	**Dominant next-state probabilities**
`Fit_Neg_Red`	Reduced dose	444	`Fit_Neg_Red`: 0.776<br>`Fit_Pos_Red`: 0.189
`Fit_Neg_Full`	Full dose	1837	`Fit_Neg_Full`: 0.572<br>`Fit_Pos_Full`: 0.396
`Fit_Pos_Red`	Reduced dose	142	`Fit_Neg_Red`: 0.558<br>`Fit_Pos_Red`: 0.394
`Fit_Pos_Full`	Full dose	1804	`Fit_Pos_Full`: 0.575<br>`Fit_Neg_Full`: 0.394
`Frail_Neg_Red`	Reduced dose	3763	`Frail_Neg_Red`: 0.796<br>`Frail_Pos_Red`: 0.195
`Frail_Neg_Full`	Full dose	2867	`Frail_Neg_Full`: 0.586<br>`Frail_Pos_Full`: 0.402
`Frail_Pos_Red`	Reduced dose	1326	`Frail_Neg_Red`: 0.573<br>`Frail_Pos_Red`: 0.417
`Frail_Pos_Full`	Full dose	3115	`Frail_Pos_Full`: 0.618<br>`Frail_Neg_Full`: 0.371

Table S6. Summary of the dominant observed transition probabilities estimated from the synthetic trajectories. For each current state and action, n denotes the total number of observed transitions for that state-action pair. Sparse or counterfactual state-action combinations were handled using smoothing in the full MDP estimation and are not shown in this summary table.
